# Evaluation of the cytotoxicity of the Bithionol - cisplatin combination in a panel of human ovarian cancer cell lines

**DOI:** 10.1186/s12885-016-3034-2

**Published:** 2017-01-13

**Authors:** Vijayalakshmi N. Ayyagari, Tsung-han Jeff Hsieh, Paula L. Diaz-Sylvester, Laurent Brard

**Affiliations:** 1 Division of Gynecologic Oncology; Department of Obstetrics and Gynecology, Southern Illinois University School of Medicine, Springfield, IL USA; 2 Center for Clinical Research, Southern Illinois University School of Medicine, Springfield, IL USA; 3 Simmons Cancer Institute at SIU, Southern Illinois University School of Medicine, Springfield, IL USA

**Keywords:** Drug combination, Cisplatin, Bithionol, Ovarian cancer cell lines, Apoptosis, Reactive oxygen species, Autotaxin

## Abstract

**Background:**

Combination drug therapy appears a promising approach to overcome drug resistance and reduce drug-related toxicities in ovarian cancer treatments. In this in vitro study, we evaluated the antitumor efficacy of cisplatin in combination with Bithionol (BT) against a panel of ovarian cancer cell lines with special focus on cisplatin-sensitive and cisplatin-resistant cell lines*.* The primary objectives of this study are to determine the nature of the interactions between BT and cisplatin and to understand the mechanism(s) of action of BT-cisplatin combination.

**Methods:**

The cytotoxic effects of drugs either alone or in combination were evaluated using presto-blue assay. Cellular reactive oxygen species were measured by flow cytometry. Immunoblot analysis was carried out to investigate changes in levels of cleaved PARP, XIAP, bcl-2, bcl-xL, p21 and p27. Luminescent and colorimetric assays were used to test caspases 3/7 and ATX activity.

**Results:**

The efficacy of the BT-cisplatin combination depends upon the cell type and concentrations of cisplatin and BT. In cisplatin-sensitive cell lines, BT and cisplatin were mostly antagonistic except when used at low concentrations, where synergy was observed. In contrast, in cisplatin-resistant cells, BT-cisplatin combination treatment displayed synergistic effects at most of the drug ratios/concentrations. Our results further revealed that the synergistic interaction was linked to increased reactive oxygen species generation and apoptosis. Enhanced apoptosis was correlated with loss of pro-survival factors (XIAP, bcl-2, bcl-xL), expression of pro-apoptotic markers (caspases 3/7, PARP cleavage) and enhanced cell cycle regulators p21 and p27.

**Conclusion:**

In cisplatin-resistant cell lines, BT potentiated cisplatin-induced cytotoxicity at most drug ratios via enhanced ROS generation and modulation of key regulators of apoptosis. Low doses of BT and cisplatin enhanced efficiency of cisplatin treatment in all the ovarian cancer cell lines tested. Our results suggest that novel combinations such as BT and cisplatin might be an attractive therapeutic approach to enhance ovarian cancer chemosensitivity. Combining low doses of cisplatin with subtherapeutic doses of BT can ultimately lead to the development of an innovative combination therapy to reduce/prevent the side effects normally occurring when high doses of cisplatin are administered.

**Electronic supplementary material:**

The online version of this article (doi:10.1186/s12885-016-3034-2) contains supplementary material, which is available to authorized users.

## Background

Drug resistance to currently used chemotherapies is the fundamental cause of recurrence and poor overall survival in ovarian cancer patients [1–3]. Between 70 and 80% of ovarian cancer patients show an initial positive response to the standard treatment (cytoreductive surgery and adjuvant paclitaxel and platinum-based chemotherapy); however, most of them will recur [4, 5]. Subsequent treatment with second-line or third-line agents (after interim non-platinum therapy) results in less than 33% response rate due to the increase of resistance to these drugs [6–9]. The poor survival rate for women with platinum-resistant ovarian carcinomas demands alternative treatment strategies.

Platinum-based chemotherapy is still an effective treatment for ovarian cancer in spite of severe side effects and development of resistance associated with its use [10]. *Cis*-diamminedichloroplatinum (II) (cisplatin) is a platinum-based compound that has clinical activity against a wide array of solid cancers including ovarian, testicular, bladder, colorectal, lung, and head and neck [11]. DNA-damage response and mitochondrial apoptosis play a major role in cisplatin’s mode of action [12, 13]. In addition, cisplatin is known to cause oxidative stress [11] via generation of superoxide anions and hydroxyl radicals [14]. Despite consistent initial responses, cisplatin treatment often results in the development of chemo-resistance, leading to therapeutic failure [10, 11]. The use of cisplatin is also limited by dose associated toxicity and side effects. Serious side effects that limit the dose of cisplatin include neurotoxicity and nephrotoxicity [11, 15]. In order to mitigate the side effects and resistance resulting from cisplatin-based chemotherapy, it is essential to investigate new drugs which are non-toxic and work in alternative/similar pathways to cisplatin, thus providing additional therapeutic options in ovarian cancer.

In recent years, a number of compounds have been explored in combination with cisplatin. Some of these include N-acetylcysteine [16], naltrexone [17], glutathione ester [18], vitamin E and losartan [19], melatonin [20], quercetin [21], metformin [22, 23], and rehmannia [24]. However, none of these combinations were successful for clinical application. In the present study, we investigated the novel combination of cisplatin with Bithionol [2, 2′-Sulfanediylbis (4, 6-dichlorophenol)] (BT) as an alternate therapeutic strategy. BT is a Food and Drug Administration-approved antiparasitic agent that has been safely dosed in humans to be used orally as a second-line medication for the treatment of helminthic infections [25]. Previously, we showed that BT exerts cytotoxic effects on a panel of ovarian cancer cell lines regardless of their cisplatin sensitivities [26]. BT half maximal inhibitory concentrations (IC_50_) observed in several ovarian cancer cell lines were well below the reported clinically tolerable levels in humans. Our recent in vivo study did not demonstrate the anti-tumor potential of BT (pharmaceutical grade); however, lack of toxicity at any of the tested doses and the ability of BT to induce apoptosis still make BT a promising candidate to be utilized in therapeutics [27]. To further explore the anti-tumor potential of BT, it is important to know the combined effects of BT with standard chemotherapeutic agent(s) such as cisplatin and paclitaxel. In this study, we assessed the antitumor efficacy of BT in combination with cisplatin using a panel of ovarian cancer cell lines such as OVACAR-3, SKOV-3 (Additional file 1) and the isogenic ovarian cancer cell lines pairs A2780 (cisplatin-sensitive) / A2780-CDDP (cisplatin-resistant) and IGROV-1 (cisplatin-sensitive) / IGROV-1CDDP (cisplatin-resistant). The primary objectives of this study are to determine the optimal combination of BT and cisplatin to achieve enhanced cytotoxic activity of both drugs and to understand the mechanism(s) of action of BT-cisplatin combination.

BT and cisplatin combinations were evaluated systematically for drug-ratio dependent interactions in vitro. The nature of the interactions between BT and cisplatin was evaluated by three different approaches – (1) sequential addition of drugs that involves pre-treatment with BT for 24 h followed by cisplatin addition (drugs in non-constant ratio), (2) simultaneous addition of both drugs in non-constant ratio and (3) simultaneous addition of drugs in constant ratio. The combination index was used to evaluate if the interactions are antagonistic, synergistic or additive.

Cisplatin and other anti-neoplastic agents exhibit cytotoxic effects via elevation of intracellular reactive oxygen species (ROS) that may contribute to their therapeutic effect. BT was shown to induce apoptosis via cell cycle regulation, ROS generation, NF-κB inhibition and autotaxin (ATX) inhibition. To investigate the molecular mechanism(s) of action of BT-cisplatin combination in ovarian cancer cells in vitro*,* we evaluated ROS generation, ATX inhibition, induction of apoptosis and expression of key apoptotic and cell cycle modulators.

## Methods

### Cell lines and chemicals

Isogenic ovarian cancer cell lines pairs, e.g., A2780 /A2780-CDDP and IGROV-1/, IGROV-1CDDP were received as a generous gift from Dr. Brodsky (Brown University, Providence, RI). The parental cell lines were purchased from Sigma and made resistant in vitro by continuous stepwise exposure to cisplatin to produce the corresponding cisplatin-resistant cell lines. All cell lines were maintained in DMEM media (Sigma) supplemented with 10% heat-inactivated FBS (Hyclone), 100 IU penicillin (Mediatech) and 100 μg/mL streptomycin (Mediatech). All cell lines were cultured at 37 °C in a humidified atmosphere at 5% CO_2_. The cisplatin-resistant variants A2780-CDDP and IGROV-1CDDP cells were treated with 3 μM cisplatin every third passage to maintain cisplatin resistance.

BT and cisplatin (*Cis*-diamminedichloroplatinum (II)) were purchased from Sigma (St Louis, MO). All primary antibodies were purchased from Cell Signaling Technologies, (Danvers, MA). PrestoBlue™ Cell Viability Reagent and ROS Dye - carboxy-H2DCFDA were purchased from Invitrogen (Carlsbad, CA).

### Cell viability assay

Cell viability after drug(s) treatment was determined by Presto Blue cell viability reagent (Invitrogen) as descried previously [26]. In brief, ovarian cancer cell lines (5 × 10^3^ cells/well) were plated into 96-well plates (Corning, Inc., Corning, NY) and incubated overnight. Cells were treated with different concentrations of both drugs either alone or in combination and incubated for 48 h. BT was tested at concentrations ranging from 3.56 μM to 100 μM and cisplatin at concentrations between 1.57 and 200 μM. A minimum of 4–6 h prior to the end of treatment, presto blue reagent was added and incubated for 48 h followed by measurement of fluorescence (540 nm excitation/590 nm emissions). DMSO concentration was corrected to 1% in all wells. All treated cells were compared against control cells (considered as 100% viable) treated with 1% DMSO media. Data were expressed as mean ± SD of triplicate experiments.

In order to determine role of ROS in BT-cisplatin induced cytotoxicity, cell viability assays were performed in the presence the antioxidant ascorbic acid (AA). Cells were pretreated with 1 mM AA for 2 h prior to addition of drugs and further incubated for 48 h. Restoration of cell viability was analyzed.

### Drug combination studies

To assess combination effects of BT with cisplatin, these drugs were combined in constant and non-constant ratios. In constant ratio combination, BT and cisplatin were combined at a fixed ratio based on the IC_50_ values of the individual drugs (i.e., concentrations causing 30–50% of cytotoxicity when these agents are used alone). Subsequently, this drug mixture was serially diluted to obtain different concentrations of the combination. The dose ranges selected for combination studies were 3.25 μM to 100 μM for BT and 1.56 μM to 200 μM for cisplatin. For non-constant ratio combination, BT and cisplatin were prepared at a series of concentrations that spans the dose–response curves for both drugs. Each concentration of BT was mixed with each concentration of cisplatin, thereby producing a matrix of multiple stock admixtures, containing both drugs together in solution at a variety of concentrations and ratios.

The nature of the interaction between BT and cisplatin was assessed using three different approaches: (1) simultaneous treatment with both drugs in non-constant ratio, where cells were treated with both BT and cisplatin simultaneously combined in a non-constant ratio; (2) simultaneous treatment with both drugs in constant drug ratio, where cells were treated with both BT and cisplatin simultaneously combined in a constant ratio and (3) pre-treatment with BT followed by addition of cisplatin in non-constant ratio, where cells were treated with different concentrations of BT for 24 h after which BT was removed and cisplatin was added for another 24 h. Here also each concentration of BT pre-treatment is followed by each concentration of cisplatin thereby producing a matrix of multiple stock admixtures (non-constant ratio).

The tumor growth inhibition obtained for BT-cisplatin combination over a range of concentrations was compared to that obtained for the individual drugs, and a measure of the synergy between the two drugs, referred to as the combination index (CI), was calculated using a median-effect mathematical algorithm [28]. CalcuSyn (BioSoft) was used to calculate CI values for drug combinations. A drug combination is synergistic if its CI value is significantly below 1; the combination is additive where the CI is between 0.9 and 1.0; and the combination is antagonistic as indicated by CI values above 1.0.

### Caspase 3/7 assay

Caspase 3/7 activity was measured using Caspase-Glo 3/7 assay kit from Promega, following the manufacturer’s instructions. Briefly, 10 × 10^3^ cells were plated per well of the 96-well plate and treated with BT and cisplatin either alone or in combination. Following treatment, Caspase-Glo 3/7 reagent was added and incubated for 30 min. at room temperature. The luminescence intensity was measured using a luminometer (luminoskan, Thermo Scientifics). Drug-treated cells were compared against cells treated with 1% DMSO media (controls). Data were expressed as mean ± SD of triplicate experiments.

### Apoptosis detection via Hoechst staining

NucBlue Live Cell Stain (Hoechst 33342; Invitrogen, Carlsbad, CA) was used to morphologically assess nuclear condensation indicative of apoptosis. This qualitative test was performed as described previously [26, 29]. In brief, cells (1 × 10^5^ cells) were seeded into 12-well plate and treated with BT and cisplatin either alone or in combination for 24 h. Following treatment, cells were washed, stained with Hoechst stain (2 drops/mL of media) for 15 min. at 25 °C and observed under a fluorescent microscope. Representative images were taken with an inverted microscope (Olympus H4-100, CCD camera) and 20× objective.

### Apoptosis quantification via TUNEL assay

DNA fragmentation was detected using the TiterTACS® 2 TdT *in Situ* Colorimetric Apoptosis Detection Kit (Trevigen, Gaithersburg, MD) following the manufacturer’s instructions. Briefly, cells were seeded at a density of 3 × 10^4^ cells/well, into 96-well flat bottom plates and incubated overnight. Cells were treated with BT and cisplatin either alone or in combination for 24 h. After treatment with drugs, cells were washed and fixed. Subsequently, labeled nucleotides were added and measurements were performed with HRP – HRP substrate (TACS-Sapphire) system. The absorbance was measured at 450 nm using a microplate reader, Multiskan (Thermo Scientifics).

### Estimation of reactive oxygen species (ROS) production

Hydrogen peroxide, hydroxyl radicals and peroxy radicals were detected via carboxy-H2DCFDA using flow cytometry as described previously [26]. Briefly, cells (1 × 10^6^) were seeded in 100 mm^2^ culture dishes and treated with BT and cisplatin either alone or in combination for 24 h. After treatment, the cells were washed once with PBS, collected by centrifugation after trypsinization, re-suspended in fresh PBS and incubated with 5 μM 5,6-carboxy-2′,7′-dichlorodihydrofluorescein diacetate (carboxy-H2DCFDA, C400, Invitrogen, Eugene, Oregon, USA) for 30 min at 37 °C. The cells were washed twice with PBS, re-suspended in an equal volume of PBS and fluorescence measured with flow cytometry. Data was acquired on a BD Accuri C6 flow cytometer and analyzed using Accuri C6 software (BD Immunocytometry-Systems, San Jose, CA). Twenty thousand cells were analyzed for each sample. Subsequent cell viability assay with AA pretreatment was performed.

### Western blot analysis

Western blotting was performed to evaluate expression of key modulators of apoptosis such as cleaved PARP, XIAP, bcl-2 and bcl-xL. Key cell cycle regulators such as p21 and p27 were also assessed by western blotting. Cell seeding, cell lysis and western botting were done as described previously [26]. In brief, cells were treated with BT and cisplatin either alone or in combination. After treatment for 24 h, cells were harvested and lysed in cell extraction buffer (Invitrogen, Carlsbad, CA) containing 10 mM Tris, pH 7.4, 100 mM NaCl, 1 mM EDTA, 1 mM EGTA, 1 mM NaF, 20 mM Na4P2O7, 2 mM Na3VO4, 1% Triton X-100, 10% glycerol, 0.1% SDS, 0.5% deoxycholate protease inhibitor cocktail and PMSF. Cell lysates were subjected to western blotting. After overnight incubation with respective primary antibodies at 4 °C, and subsequent incubation with appropriate secondary antibodies (Licor), the proteins on the blots were detected using a Licor image analyzer.

### Autotaxin (ATX) assay

The phosphodiesterase activity of ATX was measured as described previously [26]. In brief, cells were treated with BT and cisplatin either alone or in combination. Following treatment, cell-free supernatants were collected. The concentration of ATX was normalized with respect to the cell mass of samples in each well. To estimate ATX, 100 μL cell-free culture media were incubated with 100 μL substrate containing *p*-nitrophenylphosphonate (pNppp) at a final concentration of 5 mM prepared in 50 mM Tris–HCl buffer, pH 9.0. After 30 min incubation at 37 °C, the reaction was stopped by the addition of 100 μL of 0.1 M NaOH solution. The reaction product was measured by reading the absorbance at 410 nm. ATX inhibition of treated cells was calculated as the percentage of ATX activity in comparison with untreated cells.

### Statistical analysis

Comparisons between cisplatin treated and BT/cisplatin combination treated groups were performed by Student’s t–test. The significance level was set at *p* < 0.05.

## Results

### BT-cisplatin combination cytotoxicity studies

The objective of the present study is to investigate the effects of BT-cisplatin combination in ovarian cancer cell lines with special focus on cisplatin-sensitive and cisplatin-resistant isogenic pair of cell lines. Cells were exposed to different concentrations of BT and cisplatin either alone or in combination. The combination index (CI) value, calculated according to Chou’s methods [28], was used to determine the nature of the interaction between BT and cisplatin. The CI results are shown as a heat map where the green color indicates synergism (CI value < 1), the yellow color indicates additive effect (CI = 1) and the red color indicates antagonism (CI > 1). Our previous results have shown that both BT and cisplatin induced cell death in a time and dose dependent manner when added alone for 48 h (data not shown). BT-cisplatin combination-induced cytotoxicity profiles on individual ovarian cancer cell lines are described below:

#### A2780 (cisplatin-sensitive) and A2780-CDDP (cisplatin-resistant) isogenic pair

##### A2780

**Fig. 1:**
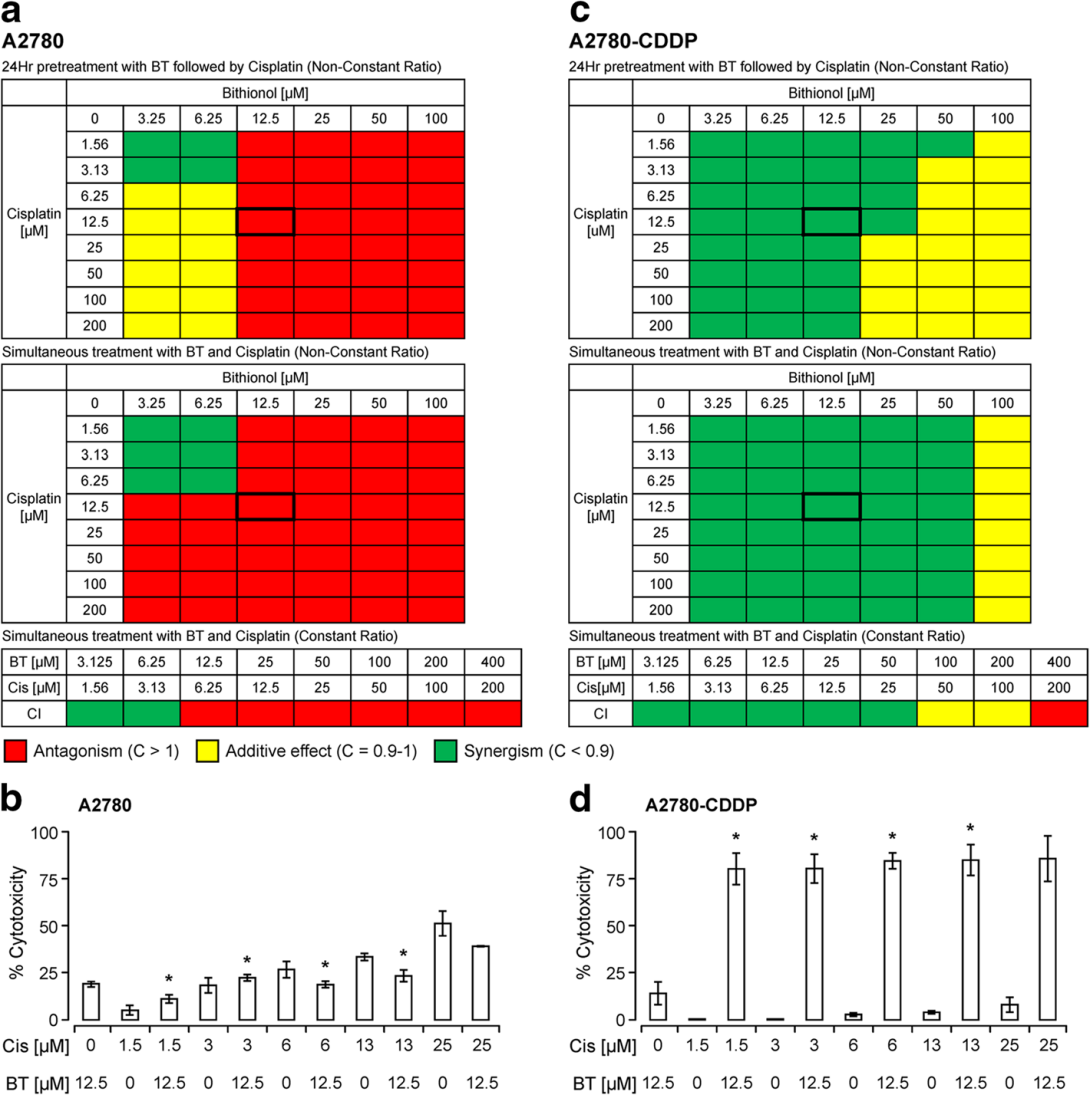
Cytotoxic potential of BT-cisplatin combination on the isogenic pair of ovarian cancer cell lines A2780 (cisplatin-sensitive) and A2780-CDDP (cisplatin-resistant). After determining viability (PrestoBlue assay) of cells treated with combinations of BT and cisplatin, combination index (CI) values were calculated and represented as heat maps where a drug combination is synergistic (green color) if CI <0.9; additive (*yellow color*) if CI is between 0.9 and 1.0; and antagonistic (red color) if CI >1.0. Combination index values for A2780 and A2780-CDDP are shown in (**a**) and (**C**) respectively. Percent cytotoxicity induced by BT/cisplatin combination at synergistic ratios for A2780 (**b**) and A2780-CDDP (**d**) are shown in bar graphs. Comparisons between cisplatin alone-treated and combination-treated for each cell line were performed by Student’s *t*-test. All data were expressed as mean ± SD of triplicate experiments. The significance level was set at *p* < 0.05 as indicated by asterisk (*)

When A2780 cells were pretreated with BT followed by cisplatin addition, there was synergy only at lower BT (3.25 and 6.25 μM) and cisplatin (1.56–6.25 μM) concentrations (Fig. 1a). At higher concentrations of cisplatin, an additive effect was observed but only lower BT concentrations (3.25–6.25 μM). Higher BT concentrations were antagonistic to cisplatin action (CI >1; represented as red). Similarly, when cells were treated with BT and cisplatin simultaneously, antagonism was observed at most drugs ratios. Synergistic effect was observed only at lower BT (3.25 and 6.25 μM) and cisplatin (1.56–6.25 μM) concentrations. As shown in Fig. 1b, combination with BT (12.5 μM) reduced the cytotoxic potential of cisplatin by 4–12% at lower cisplatin concentrations (1.56–12.5 μM). At synergistic drug ratios, combination with 6.25 μM BT enhanced cytotoxic potential of cisplatin by 4 to 33% at lower cisplatin concentrations (1.56–12.5 μM). In summary, BT and cisplatin were in general antagonistic irrespective of the drug sequence employed. However, synergy was observed only at lower BT and cisplatin concentrations, when added simultaneously or when pretreated with BT.

##### A2780-CDDP

In contrast to A2780 (cisplatin-sensitive), in A2780-CDDP (cisplatin-resistant), BT was synergistic to cisplatin action at most of the drug ratios either when cells were pre-treated with BT or when both BT and cisplatin were added simultaneously (Fig. 1c). At higher BT concentrations, additive effect was observed. When added simultaneously, at synergistic drug ratios, combination with 12.5 μM BT enhanced cytotoxic potential of cisplatin by 66 to 86% at cisplatin concentrations of 1.56–50 μM (Fig. 1d). The synergistic action of BT and cisplatin on cisplatin-resistant cell lines was independent of the drug sequence employed.

#### IGROV-1 (cisplatin-sensitive) and IGROV-1-CDDP (cisplatin-resistant) isogenic pair

##### IGROV-1

**Fig. 2:**
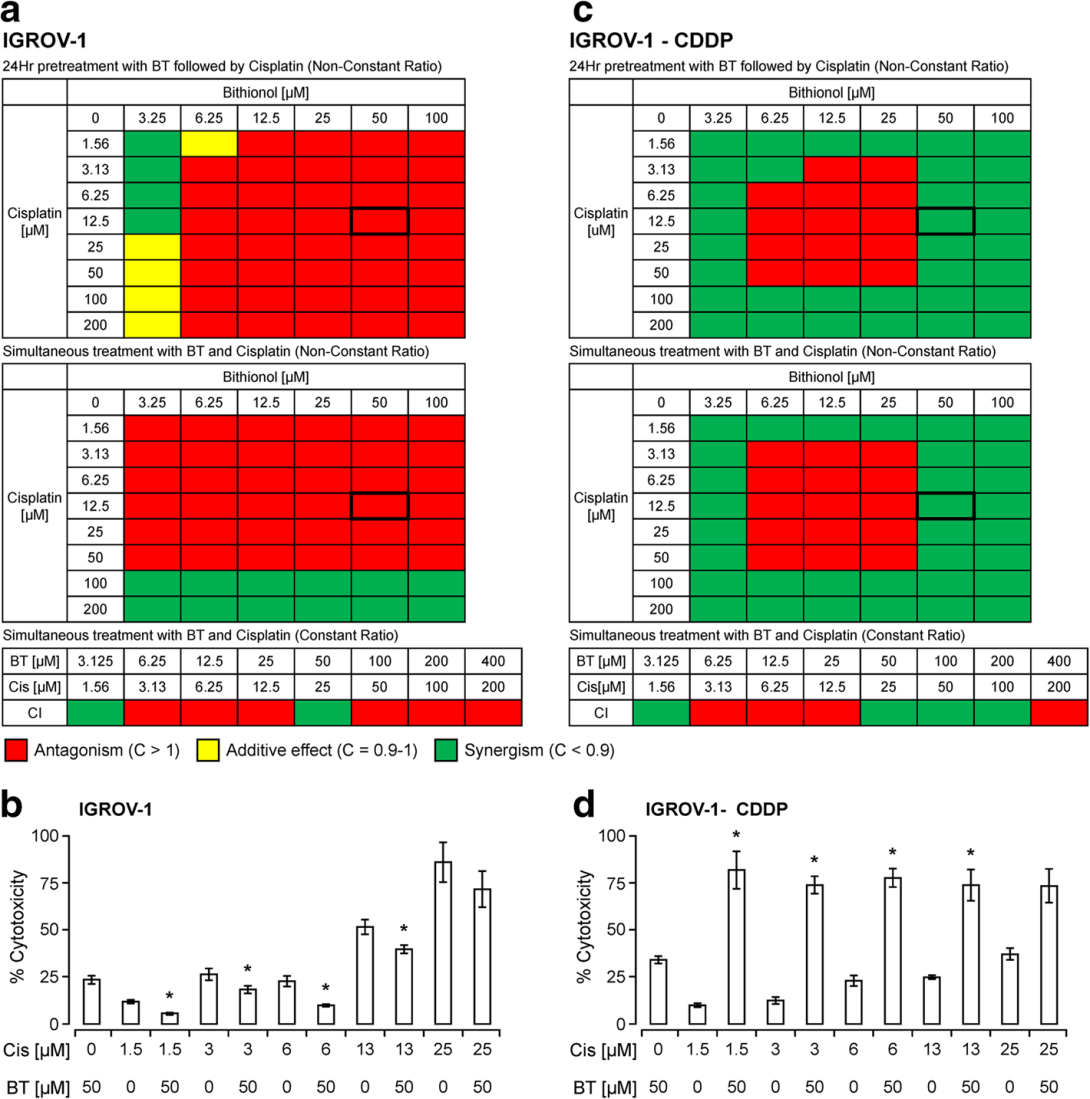
Cytotoxic potential of BT-cisplatin combination on the isogenic pair of ovarian cancer cell lines IGROV-1 (cisplatin-sensitive) and IGROV-1-CDDP (cisplatin-resistant). After determining viability (via PrestoBlue assay) of cells treated with combinations of BT and cisplatin, combination index (CI) values were calculated and represented as heat maps where a drug combination is synergistic (*green color*) if CI <0.9; additive (*yellow color*) if CI is between 0.9 and 1.0; and antagonistic (*red color*) if CI >1.0. **a** and **c** show CI values for IGROV-1 and IGROV-1-CDDP respectively. Percent cytotoxicity induced by BT/cisplatin combination at synergistic ratios for IGROV-1 (**b**) and IGROV-1-CDDP (**d**) are shown in bar graphs. Comparisons between cisplatin alone-treated and combination-treated for each cell line were performed by Student’s *t*-test. Data were expressed as mean ± SD of triplicate experiments. Asterisks (*) indicate *p* < 0.05

When IGROV-1 cells were pretreated with BT followed by cisplatin addition, antagonism was observed at most drugs ratios (Fig. 2a). Synergy only occurred at higher cisplatin (100–200 μM) concentrations, which has no physiological significance. Similarly, when these cells were treated with BT and cisplatin simultaneously, BT was synergistic to cisplatin action only at the lowest BT (3.25 μM) and cisplatin concentrations ranging 1.56 to 12.5 μM. At higher concentrations of cisplatin, an additive effect was observed but only at lowest BT concentration (3.25 μM). Higher BT concentrations were antagonistic to cisplatin action. As shown in Fig. 2b, combination with BT (50 μM) reduced the cytotoxic potential of cisplatin by 6–14% at lower cisplatin concentrations (1.56–25 μM). At synergistic drug ratios, combination with 3.25 μM BT enhanced cytotoxic potential of cisplatin by 2 to 26% at lower cisplatin concentrations (1.56–12.5 μM). In summary, the actions of BT and cisplatin on IGROV-1 cells were, in general, antagonistic. However, some synergy was observed at lowest BT and cisplatin concentrations only when pretreated with BT.

##### IGROV-1-CDDP

Interestingly, in the IGROV-1-CDDP (cisplatin-resistant) cell line, BT was synergistic to cisplatin action in a drug concentration dependent manner, either when cells were pre-treated with BT or when both BT and cisplatin were added simultaneously. Synergy was observed at low (3.25 μM) or towards higher (50 and 100 μM) concentrations of BT when combined with cisplatin at all concentrations (Fig. 2c). When added simultaneously, at synergistic drug ratios, combination with 50 μM BT enhanced the cytotoxic potential of cisplatin by 36 to 80% at cisplatin concentrations of 1.56–25 μM (Fig. 2d).

##### OVCAR-3 and SKOV-3

In these cell lines, BT and cisplatin act in general antagonistic, however, synergy was observed at very narrow drugs ratios with slightly better response when both drugs were added simultaneously (data attached as Additional file 1: Figure S1).

In summary our studies performed using isogenic pairs of cell lines show that BT and cisplatin are in general synergistic in cisplatin-resistant cell lines and antagonistic in cisplatin-sensitive cell lines. Synergy was observed when added simultaneously or when pretreated with BT. These results further support the fact that BT sensitizes cisplatin-resistant cells to respond better to cisplatin treatment.

### BT inhibits apoptosis when used in combination with cisplatin

**Fig. 3:**
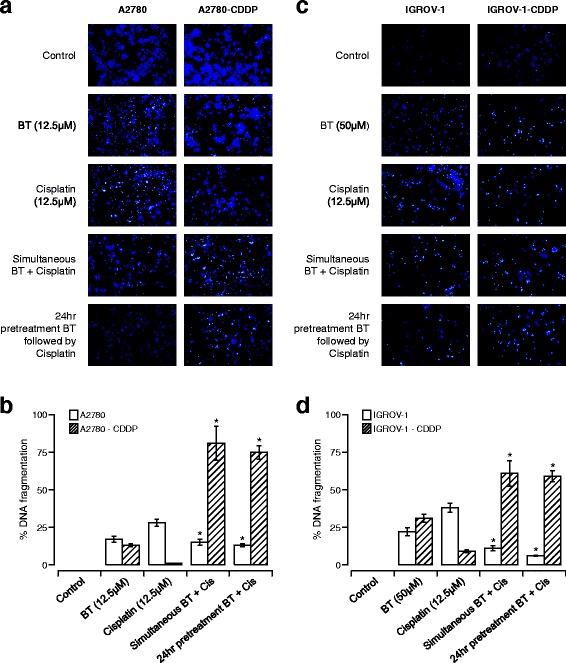
Apoptotic effects of BT-cisplatin combination on isogenic pairs of ovarian cancer cell lines. Representative images of Hoechst 33258 staining of A2780 and A2780-CDDP (**a**) or IGROV and IGROV-1-CDDP (**c**) cells treated with BT and/or cisplatin as indicated. Percent of apoptosis in terms of DNA fragmentation (quantified via TUNEL assay) are shown for A2780 and A2780-CDDP (**b**) or IGROV and IGROV-1-CDDP (**d**) cells treated with BT or cisplatin alone or in combination. Data were expressed as means ± SD of duplicate experiments. Comparisons between cisplatin alone treated and combination treated for each cell line were performed using Student’s t–test. Asterisks (*) indicate *p* < 0.05

To determine the mechanisms underlying antagonism or synergism between BT and cisplatin, we tested the effect of BT on cisplatin-induced apoptosis in cisplatin-sensitive and cisplatin-resistant isogenic pairs of cell lines. Qualitative morphological assessment was performed by nuclear (Hoechst) staining. As shown in Fig. 3a and c, vehicle-treated (control) cells stained very faintly while treated cells had a stronger blue fluorescence indicative of highly condensed chromatin, characteristic of apoptotic cells. Cisplatin-sensitive cells (A2780 and IGROV-1) treated with either BT or cisplatin alone showed higher fluorescence than those treated with BT-cisplatin combination. In contrast, cisplatin-resistant variants (A2780-CDDP and IGROV-1-CDDP) treated with BT-cisplatin combination displayed higher fluorescence than the same cell lines treated with either BT or cisplatin alone. Both isogenic pairs of cell lines exhibited similar profiles. The extent of apoptosis expressed as percentage of DNA fragmentation was quantified using the TUNEL assay. As shown in Fig. 3b and d, in both isogenic cell line pairs, cisplatin-sensitive cell lines showed considerably higher DNA fragmentation when treated with cisplatin or BT alone as compared to BT-cisplatin combination (simultaneous) treatment. A2780 cells treated with 12.5 μM BT or 12.5 μM cisplatin alone exhibited 17 ± 2 and 28 ± 3% of DNA fragmentation, respectively. When treated with both drugs in combination (simultaneously or pretreated with BT followed by cisplatin), the percentage of DNA fragmentation decreased to 15 ± 1 and 13 ± 1% respectively (Fig. 3b). For A2780-CDDP cells, treatment with 12.5 μM BT caused 13 ± 2.9% DNA fragmentation. No significant DNA fragmentation was observed when these cisplatin resistant cells were treated with 12.5 μM cisplatin alone. Upon combination, the percentage of DNA fragmentation increased significantly to 81 ± 3.76 and 75 ± 2%, respectively, when added simultaneously or pretreated with BT followed by cisplatin (Fig. 3b). IGROV-1 cells treated with 50 μM BT or 12.5 μM cisplatin alone showed 22 ± 2 and 38 ± 3% of DNA fragmentation respectively. When these cells were treated with both drugs in combination (simultaneously or pretreated with BT followed by cisplatin), the percentage of DNA fragmentation decreased to 11 ± 1 and 6 ± 1% respectively (Fig. 3d). In the case of IGROV-1-CDDP cells, treatment with 50 μM BT caused 31 ± 2.9% DNA fragmentation. No significant DNA fragmentation was observed when these cells where treated with 12.5 μM cisplatin alone. Upon drug combination, the percentage of DNA fragmentation increased significantly to 61 ± 3.76 and 59 ± 2% respectively, when drugs were added simultaneously or pretreated with BT followed by cisplatin (Fig. 3d).

### Effect of BT-cisplatin combination on apoptotic markers

**Fig. 4:**
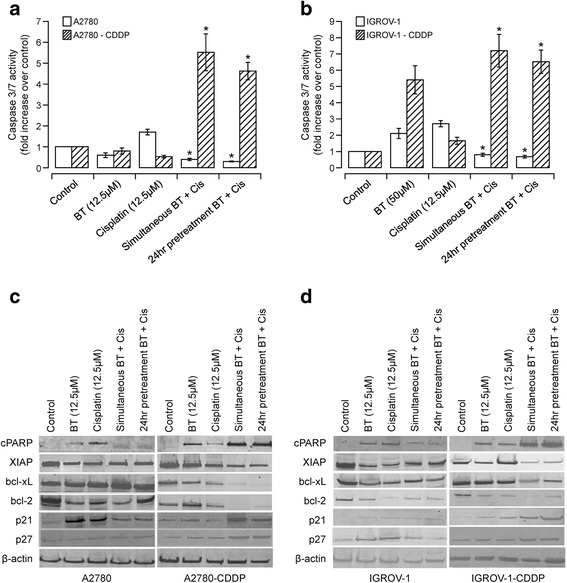
Assessment of apoptosis induced by BT-cisplatin combination on ovarian cancer cell lines. The effect of BT-cisplatin combination on caspase 3/7 activity was measured in A2780 and A2780-CDDP (**a**) or IGROV-1 and IGROV-1-CDDP (**b**) cells treated with BT or cisplatin alone or in combination. Vehicle-treated cells were considered as control against which treated cells were compared. Data were expressed as means ± SD of triplicate experiments. Comparisons between cisplatin alone-treated and combination-treated for each cell line were performed using Student’s t–test. Asterisks (*) indicate *p* < 0.05. **c** and **d** Effect of BT/cisplatin combinations on Pro-apoptotic (cPARP), anti-apoptotic (XIAP, bcl-2, bcl-xL) and cell cycle regulatory markers were assessed. Analysis of the expression of proteins in the lysates of treated and untreated A2780 and A2780-CDDP (**c**) or IGROV-1 and IGROV-1-CDDP (**d**) cells was carried out by PAGE and western blot analysis. The blots were probed with the respective primary antibodies. As an internal standard for equal loading, blots were probed with an anti-*β*-actin antibody

In order to confirm the results obtained with DNA fragmentation studies, we also assessed other apoptotic markers. As shown in Fig. 4a and b, significant reduction of caspases activity was observed when cisplatin-sensitive variants of isogenic cell line pairs, such as A2780 and IGROV-1, were treated with BT and cisplatin in combination, as compared to when treated with either of the drugs alone. In contrast, cisplatin-resistant variants of these isogenic cell line pairs (A2780-CDDP and IGROV1-CDDP) showed increased caspase 3/7 activity when treated with both drugs in combination as compared to when treated with either of the drugs alone (Fig. 4a and b).

Similarly, increased PARP protein cleavage product (85 kDa, 1 fragment) was observed in cisplatin-sensitive cell lines such as A2780 and IGROV, when treated with either of the drugs alone (Fig. 4c and d). However BT-cisplatin combination reduced the expression of cleaved PARP. In contrast, cisplatin-resistant variant of these cell lines (A2780-CDDP and IGROV1-CDDP) showed increased PARP protein cleavage product when treated with both drugs in combination as compared to when treated with either of BT or cisplatin alone.

To confirm that the potentiation/attenuation of cisplatin-induced cytotoxicity by BT treatment is accompanied by changes in the expression of key regulators of apoptosis, we also assessed the expression of XIAP, bcl-2 and bcl-xL. As shown in Fig. 4c and d, down regulation of XIAP, Bcl-2, bcl-xL was observed in cisplatin-sensitive cell lines (A2780 and IGROV-1) when treated with either of the drugs alone. However, treating the cells with BT-cisplatin combination reduced cisplatin-induced apoptotic effects as the expressions of XIAP, bcl-2 and bcl-xL increased. In contrast, cisplatin-resistant cell lines displayed significant down regulation of XIAP, bcl-2, bcl-xL when treated with BT-cisplatin combination as compared to either agent alone.

Our results suggest that BT significantly inhibits apoptosis when added in combination with cisplatin in cisplatin-sensitive cell lines (A2780 and IGROV-1) whereas it increases apoptosis in cisplatin-resistant variants (A2780-CDDP and IGROV1-CDDP). Furthermore, these results suggest that cisplatin in combination with BT caused down-regulation of key survival proteins such as XIAP, Bcl-2, and bcl-xL compared to either of the drugs alone, thus resulting in greater apoptosis/cytotoxicity. These results show that the nature of drug interactions depends on the extent of apoptosis that occurs when cells are treated in combination. Synergistic interaction enhanced apoptosis whereas antagonistic interactions reduced the extent of apoptosis.

### Effect of BT-cisplatin combination with key regulators of cell cycle

We assessed the expression of the cell cycle regulators P27 *(kip1)* and p21 in order to understand their role in causing antagonistic or synergistic effects of BT and cisplatin combination. Figure 4c and d show that BT and cisplatin both enhanced expression of P27 *(kip1)* and p21 when used alone. When cisplatin and BT were used in combination, the expression of P27 and P21 was reduced in cisplatin-sensitive cell lines (A2780 and IGROV-1) and enhanced in cisplatin-resistant cell lines (A2780-CDDP and IGROV1-CDDP), compared to cisplatin or BT treatments alone. These results are consistent with the cytotoxicity data, where combination treatment potentiated cytotoxicity in cisplatin-resistant cell lines and attenuated it in cisplatin-sensitive cell lines.

### BT potentiates cisplatin-induced apoptosis by increasing ROS generation in cisplatin-resistant cell lines whereas it reduces ROS in cisplatin-sensitive cell lines

**Fig. 5:**
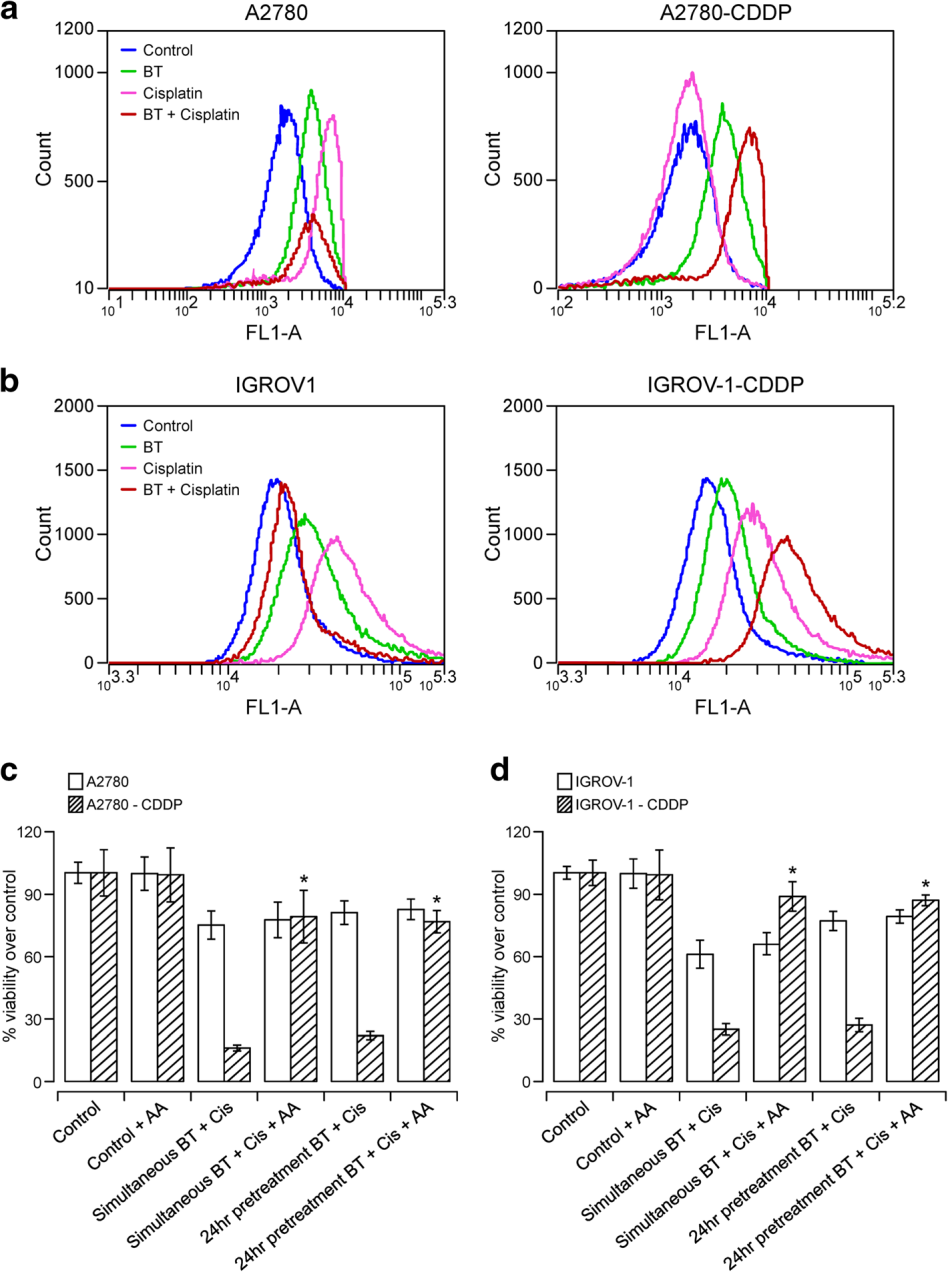
Assessment of intracellular ROS and antioxidant effect on ovarian cancer celles treated with BT cisplatin combination. Flow cytometry detection of intracellular ROS in A2780 and A2780-CDDP (**a**) or IGROV-1 and IGROV-1-CDDP (**b**) cells treated with BT or cisplatin alone or in combination. Data are presented as relative-fluorescence intensities in a 2-dimensional FACS profile (standardized gating, 20,000 events). Enhanced ROS generation is shown by shift in peaks. All experiments were performed in triplicate. **c** and **d** show the effect of the antioxidant ascorbic acid on the viability (via PrestoBlue) of A2780 and A2780-CDDP (**c**) or IGROV-1 and IGROV-1-CDDP (**d**) cells treated with BT or cisplatin alone or in combination. Control (untreated) cells were considered as 100% viable against which treated cells were compared. The results represent % viability recovery when compared with 100 μM BT-treated cells. Data were expressed as means ± SD of triplicate experiments. Comparisons between BT-cisplatin-treated in presence of ascorbic acid vs. combination-treated in the absence of ascorbic acid for each cell line were performed using Student’s t–test. Asterisks (*) indicate *p* < 0.05

We measured ROS levels to examine whether ROS are involved in the synergistic/antagonistic interaction between cisplatin and BT. As shown in Fig. 5a and b, treatment with BT or cisplatin alone lead to an increase in ROS generation evidenced by a shift in fluorescence peak (consistent with previous studies). Compared to BT, cisplatin caused greater generation of ROS in most of the cell lines. Our results further show that combinational treatment with cisplatin and BT generated more ROS relative to either of the drugs alone in cisplatin-resistant cell lines whereas it decreased ROS generation in cisplatin-sensitive cell lines. These results imply significance of ROS in the cytotoxic effects of BT-cisplatin combination. In order to confirm the role of ROS generation in antagonistic or synergistic interactions between BT and cisplatin, we tested cell viability in the presence or absence of the antioxidant ascorbic acid (AA). As shown in Fig. 5c and d, combinational treatment with cisplatin and BT in the presence of 1 mM AA restored only 10–13% viability in cisplatin-sensitive cell lines A2780 and IGROV-1. Interestingly, cisplatin-resistant variants displayed greater restoration of cell viability (75 and 85% viability restoration in A2780-CDDP and IGROV-1-CDDP, respectively). Treatment with 1 mM AA did not cause any loss in cell viability in any of the cell lines. These results implicate ROS-mediated cytotoxicity in cisplatin-resistant cell lines when treated with cisplatin and BT in combination. Lack of significant restoration of cell viability in cisplatin-sensitive cell lines implicates minimal contribution of ROS upon combination treatment.

### Effect of BT-cisplatin combination on ATX

**Fig. 6:**
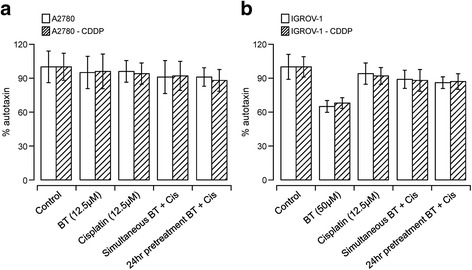
Effect of BT-cisplatin combination on ATX secretion in ovarian cancer cell lines. A2780 and A2780-CDDP (**a**) or IGROV-1 and IGROV-1-CDDP (**b**) cells were treated with drugs either alone or in combination and ATX was measured from culture media (via a colorimetric assay). The percent of ATX inhibition of treated cells was calculated using untreated cells as reference. Data were expressed as means ± SD of triplicate experiments. Comparisons between cisplatin alone-treated and combination-treated for each cell line were performed using Student’s t–test. Asterisks (*) indicate *p* < 0.05

ATX inhibition was considered major mechanism of action of BT. In order to determine if ATX inhibition potential of BT has any contribution to the synergy observed in cisplatin-resistant cell lines, we assessed the ATX levels in cell lysates treated with either of the drugs alone or in combination. As shown in Fig. 6a, no significant changes in ATX were observed when A2780 or A2780-CDDP cells were treated with either 12.5 μM BT or 12.5 μM cisplatin alone. No significant difference was seen when treated with BT-cisplatin combination also. Figure 6b shows that 12.5 μM cisplatin did not cause significant changes in ATX levels in either IGROV-1 or IGROV1-CDDP cell lines (94 ± 3 and 92 ± 5%, respectively). In contrast, treatment with 50 μM BT caused a significant decrease in ATX in both cell lines (65 and 68% respectively). However, when added in combination with cisplatin, no significant differences in ATX levels were observed as compared to cisplatin alone (89 ± 5 and 88 ± 5%, respectively). These results confirm lack of ATX involvement in drug interactions effects.

## Discussion

Treatment with of ovarian cancer with a single anticancer agent often cannot provide a satisfactory therapeutic effect in recurrent patients due to drug resistance and dose-limiting side effects [30]. Therefore, it is essential to find novel compounds/drugs that enhance the therapeutic efficiency of cisplatin, allowing for the use of lower doses and therefore reducing its toxic side effects. In this study we evaluated BT - cisplatin combination as a novel treatment for ovarian cancer.

Previously, we showed that BT exerts cytotoxic effects on a panel of ovarian cancer cell lines regardless of their cisplatin sensitivities. BT was shown to induce apoptosis both in vitro and in vivo [26, 27]. Our in vitro studies suggested that BT’s mechanism(s) of action involve cell cycle regulation, ROS generation, NF-κB inhibition and ATX inhibition. In cisplatin-resistant cell lines, ROS generation seems to be the major mechanism of BT cytotoxicity [26]. Additionally, our recent in vivo study showed that pharmaceutical grade BT was well tolerated at relatively high concentrations without any toxic side effects although an anti-tumor effect was not observed at the doses tested [27]. Any drug that is known to cause cell cycle mediated apoptosis, ROS generation or NF-κB inhibition without any toxic effects is considered an ideal candidate for combination with cisplatin for treatment of ovarian cancer. Consequently, we chose BT to be tested in combination with standard chemotherapeutic agent(s) such as cisplatin and paclitaxel.

Combination chemotherapy has been in practice for cancer treatments for many decades. The principle underlying this approach so far has been to administer each drug at their maximum tolerated dose (MTD) to achieve maximum therapeutic efficiency. However, clinical data suggested that this approach failed to exploit the full potential of many therapeutic drugs used in combinations and may have resulted in multidrug resistance in tumor cells. The major reason for such a phenomenon is exposure of tumor cells to drugs at antagonistic ratios or concentrations. The nature of the interactions (synergism or antagonism) of anticancer agent combinations against tumor cells in vitro depends on the concentrations of the individual drugs. Ratiometric dosing is an approach to develop drug combinations based on drug ratio–dependent synergy. It is essential to determine optimal drug concentrations/ratios to obtain synergy so as to prevent the exposure of cells to antagonistic ratios. In our study, we employed both constant and non-constant drugs ratios to understand the nature of interactions between our two chosen drugs. BT and cisplatin combinations were evaluated systematically for concentration-dependent interactions in in vitro using the isogenic ovarian cancer cell lines pairs A2780 (cisplatin-sensitive) / A2780-CDDP (cisplatin-resistant) and IGROV-1 (cisplatin-sensitive) / IGROV-1CDDP (cisplatin-resistant). This study shows the differential response of cisplatin-sensitive and cisplatin-resistant cell lines to BT/cisplatin combination treatment. In cisplatin-sensitive cell lines, BT/cisplatin combination was antagonistic at most drug concentrations; however the use of lower concentrations of BT and cisplatin resulted in synergy. In contrast, when tested on cisplatin-resistant cell lines, these agents were synergistic at most of the ratios/concentrations. Our results show that synergy is highly dependent on the concentration of the drugs used. Optimal response was observed when BT and cisplatin were combined at lower concentrations in a non-constant ratio. With regard to sequence of drugs applied, simultaneous treatment with BT and cisplatin appears to be a better option as compared to pretreatment with BT followed by cisplatin treatment. These results support the fact that BT sensitizes cisplatin-resistant cells to respond better to cisplatin treatment.

As reported by many, drug ratios identified as antagonistic in vitro provide inferior therapeutic activity in vivo compared with a synergistic drug ratio. Hence, it is very important to determine optimal drug ratios and then translate in vitro information on drug ratio-dependent synergy to in vivo efficacy using various drug delivery technologies. Combination drug delivery systems such as polymeric nanoparticles, dendrimers, liposomes, and water-soluble polymer-drug conjugates can be used to obtain synergistic anticancer effects while reducing concentration-related toxicity of the individual drugs [31, 32].

In addition to determining an optimal drug combination, the aim of this study was to investigate molecular mechanism(s) of action of BT/cisplatin combination in ovarian cancer cells in vitro*.* Oxidative stress and apoptosis are often the main factors contributing to the anticancer effects of drugs that target DNA [33, 34]. Since both BT and cisplatin are known to induce DNA damage, we evaluated induction of apoptosis, ROS generation, and expression of key apoptotic and cell cycle modulators as possible mechanisms of action for BT-cisplatin combination. The fact that BT has been reported to inhibit ATX activity prompted us to determine the effect of BT/cisplatin combination on ATX levels as well [26, 35, 36]. Surprisingly, when BT was added in combination with cisplatin, no significant differences in ATX levels were observed for any paired cell lines (IGROV-1 and A2780). We surmise that ATX inhibition does not play a critical role in the mechanism of action of the BT/cisplatin combination in these cell lines.

There is a consensus pointing to apoptosis as the principal mechanism by which chemotherapy agents induce cancer cell death. The majority of anticancer drugs induce apoptosis via the intrinsic (mitochondrial - cytochrome c/Apaf-1/caspase-9) pathway [37, 38]. Mitochondria are known to be both a source and a target of ROS. Disruption of mitochondrial potential can lead to oxidation of mitochondrial pores by ROS, resulting in release of cytochrome C into the cytosol [39]. The formation of an apoptosome containing cytochrome C, apoptotic protease activating factor-1 (Apaf-1) and dATP is followed by procaspase-9 recruitment and activation. Subsequently, Caspase-9 activates caspases −3 and −7 which execute the final steps of apoptosis. Consistent with previous reports on BT and cisplatin [11, 26, 27], our study shows that BT and cisplatin, when used alone, induce DNA fragmentation indicative of apoptosis. Furthermore, in cisplatin-sensitive variants, cells treated with individual drugs alone displayed higher degrees of apoptosis as compared to BT-cisplatin combination. In contrast, cisplatin-resistant variants treated with BT-cisplatin combination exhibited more apoptosis than those treated with either cisplatin or BT alone. In order to confirm the results obtained with DNA fragmentation assays, we also measured other markers of apoptosis. BT/cisplatin combination treatment reduced caspases activity and cleaved PARP in cisplatin-sensitive cell lines. In contrast, increased caspases 3/7 activity and cleaved PARP was observed in cisplatin-resistant variants of these isogenic cell line pairs treated with both drugs in combination as compared to when treated with either of the drugs alone. The nature of drug interactions depends on the extent of apoptosis that occurs when the drugs are used in combination. Synergistic interaction enhanced apoptosis whereas antagonistic interactions reduced extent of apoptosis.

Several compounds have been shown to alleviate cisplatin-induced nephrotoxicity through the translational regulation of Bcl-2 [40, 41]. Bcl-2 overexpression in conjunction with p53 has been found in fresh ovarian tissue biopsies [42]. Bcl-2 has been reported to protect ovarian cancer cells from drug-induced apoptosis, contributing to chemo-resistance [42, 43]. Inhibition of bcl-xL may increase sensitivity to chemotherapy agents such as carboplatin [44]. In this context, we also assessed the expression of pro-survival factors such as XIAP, bcl-2 and Bcl-xL. We found that in cisplatin-sensitive cell lines, BT/cisplatin combination reduced cisplatin-induced apoptotic effects as increased expressions of XIAP, bcl-2 and bcl-xL was observed. In contrast, cisplatin-resistant cell lines showed significant down regulation of XIAP, bcl-2, bcl-xL when treated with BT/cisplatin combination as compared to either agent alone, thus resulting in greater apoptosis/cytotoxicity. These observations implicate BT/cisplatin combination may be a useful approach in treating cisplatin-resistant ovarian cancers.

ROS are byproducts of normal cellular metabolism which play a crucial role in cell regulation and signal transduction [45]. Many studies have shown that cancer cells display elevated ROS levels compared to non-cancerous cells; thus, further ROS increase would make cancer cells more susceptible to oxidative damage (reviewed in [46]). Indeed, a number of common chemotherapeutic drugs including daunorubicin, cyclophosphamide, Taxol and cisplatin have been proposed to induce apoptosis through the generation of an excess amount of ROS [47–49]. However, prolonged exposure to cisplatin may reduce cellular levels of ROS resulting in chemo-resistance. Enhanced elevation of cellular ROS level by an exogenous ROS source in combination with cisplatin may re-sensitize drug-resistant cancer cells [50]. We used this approach to augment efficiency of cisplatin in cisplatin-resistant cells by combining cisplatin with BT. Previously, generation of ROS has been established as one of the underlying mechanisms of both BT and cisplatin cytotoxic activity [26, 51, 52]. Consistent with these previous reports, our study shows that treatment with either BT or cisplatin generated ROS, although ROS concentration was greater in cisplatin treated cells. Combination of cisplatin with BT further enhanced ROS production. Blocking ROS production by addition of antioxidant ascorbic acid showed a complete remission of cell death in cisplatin-resistant cells treated with BT/cisplatin suggesting that ROS production is the major mechanism of cell death for BT-cisplatin combination, thus overcoming cisplatin resistance. Our results show that BT/cisplatin combination potentiated cisplatin induced cytotoxicity by augmenting ROS accumulation and consequently activated pro-apoptosis pathways, especially in cisplatin-resistant cell lines.

Platinum derivatives have previously been shown to increase the expression of important cell cycle regulatory proteins such as p27 Kip1 and p21 Waf1/Cip1, leading to cell cycle inhibition [53, 54]. Similarly, BT was shown to cause increased expression of proteins P27 *(kip1)* and p21 in ovarian cancer cell lines in in vitro [26]. We assessed the expression of P27 *(kip1)* and p21 in order to understand the role of these cell cycle regulators in causing antagonism or synergy of cisplatin and BT combination. Our results suggest that BT reduced the magnitude of cisplatin-induced cell cycle arrest by inhibiting P27 *(kip1)* and p21 expression in cisplatin-sensitive cell lines whereas it enhanced their expression in cisplatin-resistant cell lines. With P27 *(kip1)* and p21 being key inhibitors of cell cycle, our results are consistent with the cytotoxicity data where BT-cisplatin combination treatment potentiated and attenuated cytotoxicity in cisplatin-resistant and cisplatin-sensitive cell lines, respectively.

It is known that the major drawback of current cancer chemotherapy treatments is that the use of different drugs at high doses results in undesired toxicity, drug resistance and lack of expected efficacy (possibly as a consequence of antagonistic interactions between the drugs used). As reported in this study, drugs can be synergistic at certain ratios and antagonistic at other ratios implying the need to administer them at optimal doses. Therefore, there is a need to optimize drug ratios for every combination of drug used. Our results show that the use of cisplatin and BT at lower doses enhanced the efficacy of both drugs in most ovarian cancer cell lines tested. When used in combination with BT, cisplatin can be administered at lower doses, eliminating undesired side effects without compromising its efficacy. In this context, our study provides very significant systematic evaluation of BT-cisplatin interactions for possible application in drug/cisplatin resistance ovarian cancer scenarios.

## Conclusions

Our results show that BT and cisplatin can be synergistic at certain ratios and antagonistic at other ratios implying the need to administer them at optimal doses. When used at optimal concentrations, BT/cisplatin combination potentiated cisplatin cytotoxicity by increasing the accumulation of ROS and consequently activating pro-apoptotic pathways in cisplatin-resistant cell lines. Our results suggest that repurposing of BT to be used in combination with cisplatin represents an attractive and innovative approach to enhance/restore chemosensitivity of ovarian cancer to cisplatin. Further in vivo experiments may further contribute to our understanding and confirm the therapeutic potential of BT for patients with ovarian cancer.

## Additional file

Additional file 1: Figure S1. Evaluation of the cytotoxic potential of BT-cisplatin combination against the ovarian cancer cell lines OVCAR-3 and SKOV-3. (PDF 420 kb)

## Abbreviations

ATX: Autotaxin
BT: Bithionol
IC_50_: Half maximal inhibitory concentration
IGROV-1CDDP / A2780- CDDP: Cisplatin-resistant variants of IGROV-1 and A2780
NF-κB: Nuclear factor kappa B
ROS: Reactive oxygen species
XIAP: X-linked inhibitor of apoptosis
