# Supplementary material for: Evaluation of the cytotoxicity of the Bithionol - cisplatin combination in a panel of human ovarian cancer cell lines
Source: BMC Cancer. 2017 Jan 13;17:49. doi: 10.1186/s12885-016-3034-2 (PMC5234112; doi:10.1186/s12885-016-3034-2)
Supplement: Additional file 1: Figure S1. — Evaluation of the cytotoxic potential of BT-cisplatin combination against the ovarian cancer cell lines OVCAR-3 and SKOV-3. (PDF 420 kb) [file 12885_2016_3034_MOESM1_ESM.pdf]

ADDITIONAL FILE

BT-cisplatin combination-induced cytotoxicity profiles on ovarian cancer cell lines.

OVCAR-3

BT was antagonistic to cisplatin action when cells were pretreated with BT followed by cisplatin addition (Fig. S1A). However, when BT and cisplatin were added simultaneously, a synergistic effect, highly dependent on drug concentrations was observed. When tested using a non-constant ratio or a constant ratio approach, synergy was observed near the IC_{50} concentration of BT (50 µM) when combined with lower concentrations of cisplatin (1.56 – 25 µM). At lower concentrations of BT (3.25 µM), a small additive effect was observed at lower cisplatin concentrations (3.13 – 50 µM). As shown in Fig. S1B, at synergistic drug ratios, combination with 50 µM BT enhanced the cytotoxic potential of cisplatin by almost 20 to 77% at lower cisplatin concentrations (1.56 – 12.5 µM). In summary, these results show that BT and cisplatin are in general antagonistic, however, these agents are synergistic within a very narrow range of ratios, with a slightly better response when both drugs are added simultaneously.

SKOV-3

When SKOV-3 cells were pretreated with BT followed by cisplatin, synergy was observed at low BT and cisplatin concentrations (3.25 µM and 1.56-6.25 µM, respectively) while all other concentrations resulted in antagonistic BT-cisplatin interactions (Fig. S1C). However, simultaneous addition of BT with cisplatin resulted in synergy, which was highly dependent on the concentrations of both drugs. Synergy was observed near the IC_{50} concentration of BT when combined with cisplatin at concentrations between 1.56 and 12.5 µM. At other concentrations of BT (3.25 – 25 µM), a synergistic effect was observed only at low cisplatin concentration (1.56 µM). As shown in Fig. S1D, at synergistic drug ratios, combination with 50 µM BT enhanced the cytotoxic potential of cisplatin by almost 30 to 70% at lower cisplatin concentrations (1.56 – 12.5 µM). Thus, BT and cisplatin act in general antagonistic, however, synergy was observed at very narrow drugs ratios with slightly better response when both drugs were added simultaneously.

Figure S1

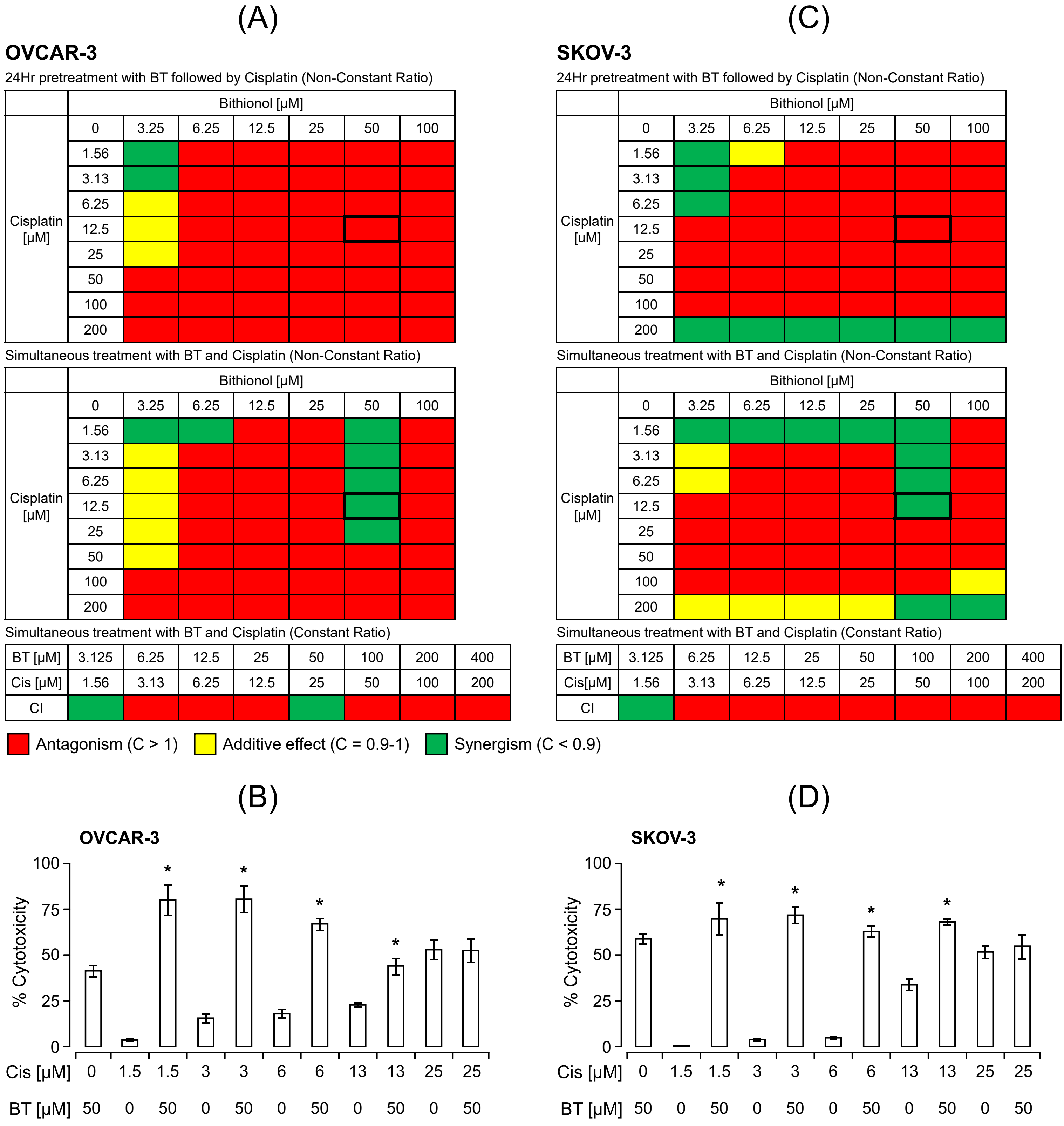

(A)
OVCAR-3
24Hr pretreatment with BT followed by Cisplatin (Non-Constant Ratio)
Bithionol [μM]
0 3.25 6.25 12.5 25 50 100
Cisplatin [μM]
1.56 3.13 6.25 12.5 25 50 100 200
Simultaneous treatment with BT and Cisplatin (Non-Constant Ratio)
Simultaneous treatment with BT and Cisplatin (Constant Ratio)
BT [μM] 3.125 6.25 12.5 25 50 100 200 400
Cis [μM] 1.56 3.13 6.25 12.5 25 50 100 200
CI
(C)
SKOV-3
24Hr pretreatment with BT followed by Cisplatin (Non-Constant Ratio)
Simultaneous treatment with BT and Cisplatin (Non-Constant Ratio)
Simultaneous treatment with BT and Cisplatin (Constant Ratio)
Cis[μM]
Antagonism (C > 1)
Additive effect (C = 0.9-1)
Synergism (C < 0.9)
(B)
OVCAR-3
(D)
SKOV-3
% Cytotoxicity
100 75 50 25 0
*
Cis [μM] 0 1.5 1.5 3 3 6 6 13 13 25 25
BT [μM] 50 0 50 0 50 0 50 0 50 0 50

Figure S1: ***Evaluation of the cytotoxic potential of BT-cisplatin combination against the ovarian cancer cell lines OVCAR-3 and SKOV-3***. After determining viability (PrestoBlue assay) of cells treated with combinations of BT and cisplatin, combination index (CI) values were calculated and represented as heat maps where a drug combination is synergistic (green color) if CI < 0.9; additive (yellow color) if CI is between 0.9 and 1.0; and antagonistic (red color) if CI > 1.0. CI values of OVCAR-3 and SKOV-3 are shown in **(A)** and **(C)** respectively. **(B and D**) % cytotoxicity induced BT/cisplatin combination at synergistic ratios of OVCAR-3 and SKOV-3 respectively. Percent cytotoxicity induced by BT/cisplatin combination at synergistic ratios for OVCAR-3 **(B)** and SKOV-3 **(D)** are shown in bar graphs. Comparisons between cisplatin alone-treated and combination-treated for each cell line were performed by Student's t-test. All data were expressed as mean ± SD of triplicate experiments. The significance level was set at $p < 0.05$ as indicated by asterisk (*). Human ovarian carcinoma cell lines, OVCAR-3, SKOV-3 were provided by Dr. McAsey (SIU School of Medicine, Springfield, IL). The significance level was set at $p < 0.05$ as indicated by asterisks (*).
